# Wine Pomace Product Inhibit *Listeria monocytogenes* Invasion of Intestinal Cell Lines Caco-2 and SW-480

**DOI:** 10.3390/foods10071485

**Published:** 2021-06-26

**Authors:** Gisela Gerardi, María D. Rivero-Pérez, Mónica Cavia-Saiz, Beatriz Melero, Alicia Salinero-Zorita, María L. González-SanJosé, Pilar Muñiz

**Affiliations:** Department of Biotechnology and Food Science, Faculty of Sciences, University of Burgos, Plaza Misael Bañuelos, 09001 Burgos, Spain; mggerardi@ubu.es (G.G.); drivero@ubu.es (M.D.R.-P.); monicacs@ubu.es (M.C.-S.); bmelero@ubu.es (B.M.); asz1001@alu.ubu.es (A.S.-Z.); marglez@ubu.es (M.L.G.-S.)

**Keywords:** *Listeria monocytogenes*, Caco-2, SW480, virulence, red wine pomace, E-cadherin, occludin, claudin, polyphenols

## Abstract

Red wine pomace products (WPP) have antimicrobial activities against human pathogens, and it was suggested that they have a probable anti-*Listeria* effect. This manuscript evaluates the intestinal cell monolayer invasive capacity of *Listeria* monocytogenes strains obtained from human, salmon, cheese, and *L. innocua* treated with two WPP (WPP-N and WPP-C) of different polyphenol contents using Caco-2 and SW480 cells. The invasion was dependent of the cell line, being higher in the SW480 than in the Caco-2 cell line. Human and salmon *L. monocytogenes* strains caused cell invasion in both cell lines, while cheese and *L. innocua* did not cause an invasion. The phenolic contents of WPP-N are characterized by high levels of anthocyanin and stilbenes and WPP-C by a high content of phenolic acids. The inhibitory effect of the WPPs was dependent of the strain and of the degree of differentiation of the intestinal cells line. The inhibition of *Listeria* invasion by WPPs in the SW480 cell line, especially with WPP-C, were higher than the Caco-2 cell line inhibited mainly by WPP-N. This effect is associated with the WPPs’ ability to protect the integrity of the intestinal barrier by modification of the cell–cell junction protein expression. The gene expression of E-cadherin and occludin are involved in the *L. monocytogenes* invasion of both the Caco-2 and SW480 cell lines, while the gene expression of claudin is only involved in the invasion of SW480. These findings suggest that WPPs have an inhibitory *L. monocytogenes* invasion effect in gastrointestinal cells lines.

## 1. Introduction

Microbial cross-contamination is a problem in the food industry, and listeriosis outbreaks are a focus of special interest [1,2]. *Listeria* monocytogenes is a Gram-positive pathogenic bacterium, responsible for serious systemic infections such as septicemia, gastroenteritis, and meningitis, among others, both in animal species and in humans, with a high lethality rate [3,4]. According to the last report published by the European Food Safety Authority (EFSA) and the European Centre for Disease Prevention and Control (ECDC), listeriosis was the fifth-most frequent zoonoses but the most severe one, with 229 deaths in Europe in 2018 (EFSA and ECDC 2019). The control of *L. monocytogenes* is difficult due to its ability to tolerate a wide range of temperatures (−1.5–45 °C) and pH (4.3–9.1), salt concentration, and lower water activity. Furthermore, it is a facultative microorganism, resistant to alkaline media and to preservatives, such as sodium nitrate [5,6]. Due to these characteristics, it can be present in a wide range of foods, as well as survive various processing technologies. The cell wall of this type of bacteria forms a rigid exoskeleton, with the surface proteins mediating its contact with the host cells [7], associated with the ability of intracellular bacterial survival and propagation cell to cell. The proteins Internalin A and B (InlA and InlB), encoded by the genes *inlA* and *inlB*, respectively, are the main invasion factors, which bind to the receptors present on the surface of the host cell E-Cadherin and Met, allowing adherence and, therefore, the invasion of bacteria after the destabilization of the cell membrane [8,9].

To prevent microbial cross-contamination and increase foodstuff safety and shelf lives, the food industry uses antimicrobial agents—commonly, synthetic or “chemical” additives. However, in the last decade, the demand for using natural products with antimicrobial properties has increased. The activity of natural products derived from plants against microorganisms has been mainly associated with the presence of phenolic compounds with antimicrobial activities [10,11]. Phenolic compounds can act on the microbial cell membrane in the lipid bilayer, causing de-arrangement in membrane structures or interacting with soluble extracellular proteins, inducing bacterial cell death [12,13,14].

Wine grapes are very rich in phenolic compounds, and most of them are not extracted during the winemaking process. Thus, the wine industry generates large amounts of grape pomace rich in phenolic compounds that have been considered as an alternative for obtaining sustainable functional ingredients, which may be used in the food industry with different purposes [15,16,17], antimicrobial actions against foodborne pathogens being one of them [18]. The antimicrobial activity of wine pomace compounds to inhibit the pathogenic activity of foodborne pathogens such as *L. monocytogenes* has been described in previous works [16,19]. The anti-*Listeria* activity could be attributed to the wine pomace flavonoids that interact with soluble extracellular proteins and the bacterial cell wall [19,20,21]. In addition, wine pomace prevents epithelial dysfunction and changes of permeability through the improvement of adheren junctions (E-cadherin) and tight junctions (occludin and claudin) via NF-κB pathway downregulation, which represent the mechanisms involved in *L. monocytogenes* intestinal invasion [22,23,24].

Wine pomace products that have antivirulence effects on different strains of *Listeria* monocytogenes could then be used as a functional ingredient in the food industry. Therefore, we investigated the invasion of *L. monocytogenes* strains treated with WPP using Caco-2 and SW480 intestinal cell lines and the expression of adheren and the tight junctions involved in the mechanism of invasion. Our hypothesis is that wine pomace products that have antimicrobial activity in vitro might also have invasion potential capacity against different *L. monocytogenes* strains.

## 2. Materials and Methods

### 2.1. Chemicals

Saline peptone water, Brain Heart Infusion (BHI), Tryptic Soy Broth (TSB) supplemented with Yeast, Plate Count Agar (PCA), Tryptone Sulphite Neomycin (TSN), Dextrose Sabouraud (DS), and Mannitol Egg Yolk Polymyxin (MYP) mediums were obtained from Merck KGaA, (Darmstadt, Germany). Acetonitrile; amphotericin B solution (250 μg/mL); 2,2′-Azinobis 3-ethylbenzothiazoline-6-sulphonic acid (ABTS); Fetal Bovine Serum (FBS); glacial acetic acid; hydrochloric acid; 6-hydroxy 2,5,7,8-tetramethyl-2-carboxylic acid (Trolox); L-glutamine solution (200 mM); MEM (Minimum Essential Medium); methanol; penicillin (10,000 U/mL); pure phenolic compounds (caffeic acid, catechin, epicatechin, epicatechin gallate, ethyl gallate, gallic acid, gentisic acid, p-coumaric acid, procyanidin B1, procyanidin B2, protocatechuic acid, syringic acid, t-resveratrol, t-piceid, and vallinic acid); streptomycin solution (P/S; 100 mg/mL); and 2,4,6-Tris (2-pyridyl)-S-triazine (TPTZ) were purchased from Sigma-Aldrich Chemical Co. (St. Louis, MO, USA). Folin–Ciocalteu (FC) reagent, 70% (*v*/*v*), iron (III)-chloride acid (FeCl3), iron (II)-sulphate (FeSO_4_), sodium acetate (NaC_2_H_3_O_2_), and sodium carbonate (Na_2_CO_3_) were purchased from Panreac Quimica S.L.U. (Barcelona, Spain). Malvidin-3-glucoside was obtained from Estrasynthese (Genay, France).

### 2.2. Wine Pomace Products (WPP)

Red wine pomaces were collected, from the same vintage, in two different wineries of two Spanish wine regions, one cited in the north and other in the center of Spain. Both wineries made red wines mainly with *Vitis vinífera* var. Tempranillo grapes. The use of wine pomaces from different regions and wineries was made with the intention of considering the intrinsic variability of wine pomaces to obtain more generalizable or extrapolated results. The WPP used in this study, WPP-N (red wine pomace product from the north winery) and WPP-C (red wine pomace product from the center winery), were made by applying previously described processes with minor modifications. Briefly, each collected wine pomace was dehydrated in an oven at 60 °C. Each WPP was made from seedless dried wine pomaces by milling and sieving (particle size <0.250 mm), and the microbiological stabilization was done by heat treatment (90 °C/90 min).

### 2.3. WPP Microbiological Quality

One gram of each one of the WPP (N and C) was added to 9 mL of different culture media (saline peptone water, BHI, and TSB-Y) and incubated for 24 h at 37 °C. To determine the total viable counts (TVC) and *Clostridium* spp., 1 mL was poured on PCA and TSN, respectively, and incubated at 30 °C for 72 h for TVC and 37 °C for 24 h in anaerobic conditions for *Clostridium* spp. One hundred microliters were streaked on DS and MYP to determine the molds and yeast and *Bacillus* spp., respectively, and incubated at 25 °C for 5 days for the molds and yeast and 30 °C during 24 h for *Bacillus* spp. Microbiological assays were carried out in triplicate.

### 2.4. Characterization of Phenolic Compounds

Extractable phenolic compounds of each WPP were quantified by HPLC after extracting using acidified methanol. Rehydrated powders were extracted with methanol/HCl (97:3) at 25 °C for 24 h and continuous stirring. Then, supernatants were decanted and filtered through a VWR cellulose filter with 8–12-µm particle retention (VWR International BVBA, Leuven, Belgium). Each supernatant was raised to a known previous volume to be analyzed. Extracts were made in triplicate. Different phenolic compounds were analyzed using analytical reverse-phase HPLC on an Agilent 1100 series HPLC system (Agilent Technologies Inc., Palo Alto, CA, USA) coupled to a diode array detector. Hydroxybenzoic acids, hydroxycinnamic acids, flavan-3-ols, and stilbenes were analyzed using a spherisorb3 ODS2 reverse-phase C18 column (250 mm × 4.6 mm, 3-μm particle size, Waters Cromatografia S.A., Barcelona, Spain) using the chromatographic conditions previously published [25,26]. Retention times and diode array spectral of each peak were compared with the respective standards, which was useful to identify each peak. The results were expressed in µg/g WPP using the corresponding calibration curves. Anthocyanins were analyzed using a Nova-Pak reverse-phase C18 column (300 mm × 3.9 mm, 4-µm particle size, Waters Ltd., Elstree, UK). The eluent was monitored at 520 nm, with compound spectra obtained between 220 and 600 nm. Peak identification was performed by comparison of the retention times and diode array spectra of the standards and our own library. The results were expressed as µg/g WPP of the major extracted anthocyanin that was malvidin-3-glucoside.

### 2.5. Quencher Antioxidant Capacity

QUENCHER (Q-) methods better reproduce the real circumstances in which WPP solid products, and not their extracts, are used. Q-methods are based on the corresponding classical methods, but they were adapted to measure the TAC (total antioxidant capacity) of solid products. QUENCHER versions of three classical TAC assays: Folin–Ciocalteu index, ABTS (2,2ʹ-Azino-bis(3-ethylbenzthiazoline-6-sulfonic Acid), and FRAP (Ferric Reducing Antioxidant Power), were selected to evaluate the TAC of the WPP [27]. QUENCHER Folin–Ciocalteu assay (Q-FC), QUENCHER ABTS assay (Q-ABTS), and QUENCHER FRAP assay (Q-FRAP) results were expressed, respectively, as µmol gallic acid equivalents (GAE), µmol Trolox equivalents (TE), and µmol of Fe(II) per g of WPP. All measurements were performed in triplicate.

### 2.6. Bacteria Strains, Culture Conditions, and Treatment with WPP

The *L. monocytogenes* strains used in this study were either previously isolated from food products in the food technology area at the University of Burgos (cheese—E10.652, salmon—S11, and a *L. innocua* strain from meat) or from human cases (ILSI9, ILSI17, and ILSI18) obtained from the International Life Sciences Institute (ILSI) North America *Listeria* monocytogenes strain collection [28]. *L. monocytogenes* PCR-serogrouping was performed for the salmon-derived strain using a multiplex PCR as previously described [29], using ILSI strains as the controls. The cheese-derived strain was previously characterized by some of the authors [30]. Bacterial cultures were maintained at −80 °C in BHI containing 30% glycerol. Each *Listeria* strain was grown on Tryptic Soy Broth supplemented with yeast extract (TSB-Y) at 37 °C overnight to obtain 9 log cfu/mL. Then, the culture was diluted to inoculate 7 log cfu/mL in BHI supplemented with 40 g/L of each WPP (WPP-N and WPP-C) and incubated for 24 h and 48 h at 37 °C. The quantity of WPP supplemented was decided considering previous satisfactory results [15]. To consider the possible effect of the medium pH, which was modified by the presence of the WPP, a control with modified pH (with sterile tartaric acid) was also included. A previous trial incubating controls for 24 h and 48 h at 37 °C was also performed to optimize the incubating time to further in vitro virulence assays. All assays were performed in triplicate.

### 2.7. In Vitro Virulence Assays

For the in vitro virulence assay, human adenocarcinoma cell lines Caco-2 and SW480 from ECACC (European Collection of Authenticated Cell Cultures, Salisbury, UK) were used, and the assays were performed as previously described [31]. Caco-2 and SW480 cells were cultivated in a MEM (Minimum Essential Medium), which contained 2-mM L-glutamine, 20% FBS (Fetal Bovine Serum), 100-mg/mL streptomycin sulfate, 1% PAA (nonessential Amino Acid Solution 100x), and 0.5-mg/mL amphotericin B at 37 ᵒC in a humidified incubator containing 5% CO_2_. Cell monolayers were infected with the different strains of *L. monocytogenes* with and without a previous treatment with WPP-N and WPP-C, and a multiplicity of infection (MOI) of 25:1 was used. After 1 h of incubation at 37 °C, the cell monolayers were washed with PBS and incubated in MEM and 10% of fetal bovine serum, containing gentamicin (100 µg/mL) for 4 h (intracellular growth). Half of the samples were lysed with 1 mL of 0.1% Triton X-100, and counting was performed by plating on TSAYE agar. The other half of the samples were tripsinized and centrifuged, and the pellets were frozen until the q-PCR analysis. All assays were independently repeated four times.

### 2.8. Analysis of Adherent and Tight Junctions by Quantitative Real-Time PCR (q-PCR)

Caco-2 and SW480 pellets were resuspended in TRI Reagent solution (Applied Biosystems, Foster City, CA, USA). After treatment with DNase I (Thermo Fisher Scientific, Inc., Waltham, MA, USA), 1 µg of total RNA was reverse-transcribed using a First Strand cDNA Synthesis kit (Thermo Fisher Scientific), and, finally, amplified using an iQ™ SYBR^®^ Green Supermix (Bio-Rad Laboratories, S.A., Madrid, Spain). All the procedures were performed according to the manufacturers’ protocols. The sequences of the primer sets (forward and reverse) were, glyceraldehyde-3-phosphate dehydrogenase (GAPDH), 5′-GCTCTCCAGAACATCATCCC-3′ and 5′-GTCCACCACTGACACGTTG-3′; claudin, 5′-GTGGAGGATTTACTCCTATGCCG-3′ and 5′-TCAAGGCACGGGTTGCTT-3′; E-cadherin, 5′-ATGCTGAGGATGATTGAGGTGGGT-3′ and 5′-CAAATGTGTTCAGCTCAGCCAGCA-3′; and occludin, 5′-TCAAACCGAATCATTATGCACCA-3′ and 5′-AGATGGCAATGCACATCACAA-3′. Amplification efficiencies were calculated for each pair of primers, and efficiency-corrected quantification was performed using 2^−∆∆Ct^ with GAPDH as the reference gene. Relative gene expression was expressed as the folds of change compared to the control (control cells uninfected with *L. monocytogenes*).

### 2.9. Statistical Assays

Representative data were expressed as the mean ± standard deviation of independent experiments. Statistical analysis was performed using Statgraphics^®^ Centurion XVI, version 16.2.04 (Statpoint Technologies, Inc., Warrenton, VA, USA). ANOVA analysis and the Student’s *t*-test were used to determine the statistical significance, with a *p*-value of <0.05).

## 3. Results and Discussion

The antioxidant and antimicrobial properties of wine pomace products (WPP) are of interest to the food industry, and they confer the possibility of considering the role of WPP as food preservatives. In previous studies, it was observed that the WPP obtained from red wine pomace could be used as natural alternative additives due to their antioxidant activity, their ability to improve microbial stability, inhibiting spoilage bacteria growth in foods with low contents of salt [19], and their bactericide capacity against *L. innocua*, showed in vitro [15].

In this study, we evaluated the possible association between the bactericide effect of WPP observed previously and their antivirulence effect in intestinal cells. Intestinal epithelial cells are the first line of defense against pathogens that can breach this barrier. The implication of cell junction proteins (adherens and tight junctions) is that they are critical for the maintenance of the intestinal barrier. Therefore, this study evaluated the intestinal invasion of *L. monocytogenes* treated with two types of WPP, together with their effect on intestinal E-cadherin as an InlA cell receptor and on tight junction proteins occludin and claudin for their role in the maintenance of intestinal paracellular permeability.

Before studying the WPP effect in *L. monocytogenes* virulence, and to avoid contamination from possible microorganisms contained in WPP, the microbiological quality of each WPP was evaluated. The results revealed that both WPP (N and C) did not show any microbial load.

Furthermore, extractable phenols were analyzed as the principal antimicrobial agents of WPP, and the antioxidant capacities were considered as an index of additional interesting properties of the WPP also associated to the phenol content.

The levels of the different phenolic compounds analyzed (Table 1) showed significant differences between WPP.

The largest differences were detected between the anthocyanin levels, which were significantly higher in WPP-N than in WPP-C (1225 ± 98 and 201 ± 13, respective global levels). On the contrary, the levels of the phenolic acids (hydroxybenzoic and hydroxycinnamic acids) were statistically higher in WPP-C than in WPP-N. The total content of the flavan-3-ols was similar in both WPP, but stilbenes, like anthocyanins, were statistically higher in WPP-N than in WPP-C. These results evidenced the possible large variability that can occur between the compositions of the wine pomaces collected in different regions and wineries, which derived from pedologic-climatic conditions, cultural vine practices, and oenological processes, among other factors.

**Figure 1:**
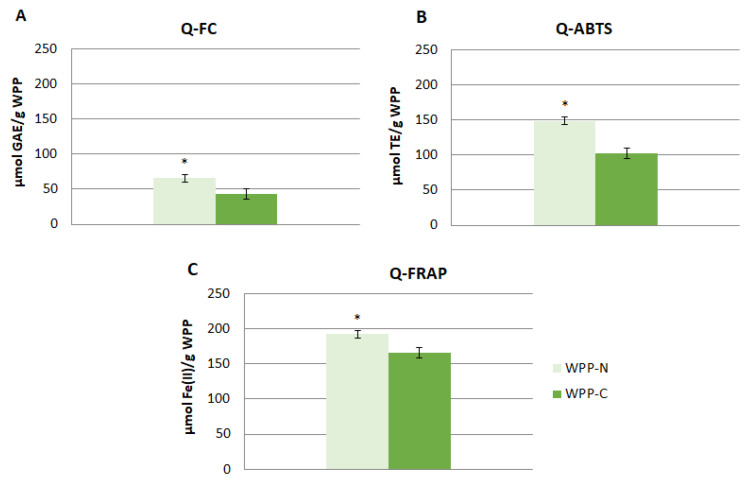
Total antioxidant capacity of the studied wine pomace products (WPP) evaluated using the Quencher method (Q-) of the classical Folin–Ciocalteu (FC) (**A**), Q-ABTS (**B**), and Q-FRAP (**C**) assays. GAE: gallic acid equivalents; TE: Trolox equivalents. WPP-N: Wine pomace product of the north winery; WPP-C: Wine pomace product of the center winery. Results are expressed as the mean ± standard deviation (*n* = 3). * Indicates statistical higher values (*p* < 0.05).

The results of the total antioxidant capacity (TAC) of both WPP also highlighted the differences between the products (Figure 1). The Q-FC results showed higher values in WPP-N than in WPP-C, agreeing with the results of the individual phenolic compounds. Similarly, the Q-ABTS and Q-FRAP values were higher in WPP-N, probably due to its higher content of polyphenol compounds [32,33].

### 3.1. Effect of WPP on the Invasive Capacity of Listeria monocytogenes in Caco-2 and SW480 Cell Lines

*L. monocytogenes* can cause two forms of listeriosis: noninvasive gastrointestinal listeriosis and invasive listeriosis [1,2]. The invasive listeriosis can be studied in vitro, based on the ability of this microorganism to enter human intestinal cells. In this study, the invasive capacity of diverse *L. monocytogenes* strains isolated from humans (ILSI9, ILSI17, and ILSI18); salmon (S11); and cheese (E10.652), together with one *L. innocua* strain, was evaluated after their serotyping.

The serotyping results showed that the salmon strain belonged to the serogroup 1/2a, 3a, as well as the cheese strain and ILSI18. ILSI9 belonged to the serogroup 1/2b, 3b, 7 and ILSI17 to the 1/2c, 3c serogroup. It is well-known that serogroups 1/2a, 3a and 1/2c, 3c are related with the food environment, and 1/2b, 3b, 7 and 4b, 4d, 4e are related with human cases and outbreaks. Besides, the 1/2a, 1/2b, and 4b serotypes are responsible for 98% of listeriosis cases [34].

The invasion capacity of the *Listeria* strains was evaluated using two cell lines: Caco-2 and SW480, with different cell structures and growth rates. The Caco-2 cell line is a model of differentiated cells that retain their morphology and most of the enterocytes function, resembling the intestinal epithelium [35]. The SW480 cell line is a model of moderately differentiated cells and with a high invasive capacity [36]. Cell adhesion is critical in the organization of epithelial cells—in particular, in the establishment of the adheren junctions mediated by the presence of E-cadherin, which initiates a cascade of events involved in the regulation of the junction complex and cellular polarization [37]. Changes in these junctions lead to increases of the membrane permeability and the virulence of pathogens.

It is known that the virulence of *L. monocytogenes* is due to the capacity to adhere, invade, and multiply within cells, adhesion to the intestinal epithelium being the first step in the pathogenesis that precedes the invasion, and consequently, the inhibition of adhesion can prevent colonization and can limit opportunities for systemic infection [38]. Therefore, the number of ingested bacteria is not the only important determinant for the development of listeriosis, and *Listeria* strains’ invasive potential must also be considered [39].

The invasive capacity of the different *Listeria* strains was evaluated at two times of bacterial growth (24 and 48 h). Monolayer cultures of the Caco-2 and SW480 cells were inoculated with each *Listeria* strain cultured for 24 and 48 h. The results of the invasion produced by the different strains were expressed in cfu/mL after the lysis of the host cells. The obtained results showed the invasive capacity of ILSI9, ILSI7, ILSI18, and S11 at 24 h and 48 h in both cell lines, while E10.652 and *L. innocua* did not invade in either cell line (Table 2 and Table 3). These results agree with previous studies that established that the capacity of *L. monocytogenes* to adhere to intestinal cells varies widely depending on the *Listeria* strain [40,41]. The obtained results revealed that the serogroups tested were able to invade both cell lines, although differences within serogroup 1/2a, 3a were found.

Previous works [42,43] also noted differences in serogroup 1/2a, 3a strains isolated from a poultry processing plant. In these studies, one out of two isolated strains from serogroup 1/2a, 3a, belonging to the same sequence type (ST9), was not able to invade Caco-2 cells. This also occurred with two out of three isolate strains from serogroup 1/2c, 3c and ST121, although, in both STs, truncation in Internalin A (InlA) was found. Furthermore, Ciolacu et al. [44] showed that some strains from serogroup 1/2a, 3a, as well as a strain from the 1/2c, 3c serogroup, were also not able to invade and noted the presence of mutations in InlA as a possible reason to explain the inability to invade.

In general, the invasion of *L. monocytogenes* strains cultured for 24 h was statistical significantly higher than those grown 48 h (Student’s *t*-test, *p* < 0.05) and was higher in the SW480 than in the Caco-2 cell line (Table 2 and Table 3). The data showed significant differences (Student’s *t*-test, *p* < 0.05) among the invasion capacity of each *Listeria* strain, which were more numerous in the case of the more differentiated cell model, the Caco-2 cell line. ILSI17 was the most invasive *Listeria* strain in the two cell lines.

The difference in the *L. monocytogenes* invasive capacity observed between both intestinal lines could be a consequence of the different characteristics of each type of intestinal cell line. The Caco-2 cell line exhibits characteristics of enterocytes with a polarized epithelium with a homogenous distribution of microvilli. However, SW480 cells correspond to nondifferentiated cells, and their surface protein expression differs from the Caco-2 cells with irregular microvilli and with high losses of the tight junction, indicative of highly disorganized epithelia [45]. Furthermore, other authors observed that *Listeria* Adhesion Proteins (LAP) binding Hsp60 activates NF-κB signaling in Caco-2 cells, facilitating the myosin light-chain kinase (MLCK)-mediated opening of the epithelial barrier via the cellular redistribution of the tight junction proteins (claudin-1 and occludin) and adheren junction protein (E-cadherin) [24]. E-cadherin can bind Internalin A (InlA) of the *Listeria* surface [46], and this capacity is essential for the adhesion and penetration of *L. monocytogenes* into the intestinal barrier [47]. Therefore, the expression levels of tight junction proteins (TJ) claudin and occludin are involved in the maintenance of intestinal paracellular permeability and the expression of E-cadherin implied in crossing the intestinal epithelial barrier and were studied by real-time PCR in both Caco-2 and SW480 cells after *L. monocytogenes* incubation using *L. monocytogenes* strains.

**Figure 2:**
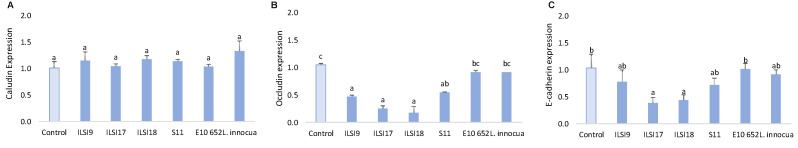
Caco-2 cell junction protein expression after *L. monocytogenes* invasion. Values represent the relative gene expression of claudin (**A**), occludin (**B**), and E-cadherin (**C**) of the control cells (cells uninfected with *L. monocytogenes*) and cells infected with ILSI9, ILSI17, and ILSI18 (clinical *L. monocytogenes* strains) and S11 and E10.652 (food *L. monocytogenes* strains) and *L. innocua*. The results are presented as the means ± SD (*n* = 4). Different letters on each column indicate significant differences (ANOVA, *p* < 0.05) among the mean values.

**Figure 3:**
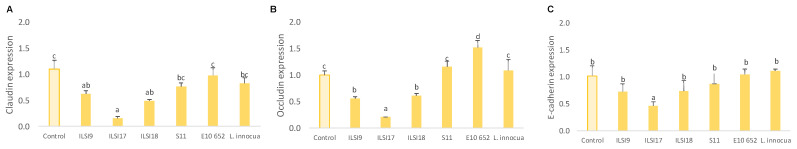
SW480 cell junction protein expression after *L. monocytogenes* invasion. Values represent the relative gene expression of claudin (**A**), occludin (**B**), and E-cadherin (**C**) of the control cells (cells uninfected with *L. monocytogenes*) and cells infected with ILSI9, ILSI17, and ILSI18 (clinical *L. monocytogenes* strains) and S11 and E10.652 (food *L. monocytogenes* strains) and *L. innocua*. The results are presented as the means ± SD (*n* = 4). Different letters on each column indicate significant differences (Student’s *t*-test, *p* < 0.05) among the mean values.

The noninvasive *Listeria* strains (E10.652 and *L. innocua*) did not induce any changes in the expression of TJ proteins (claudin and occludin) or of the adheren (E-cadherin) compared with the noninfected cells (control) (Student’s *t*-test, *p* < 0.05). This fact can explain the incapacity of these strains to invade the cells (both Caco-2 and SW480) (Figure 2 and Figure 3). However, the other studied strains, ILSI17, ILSI18, ILSI9, and S11, which were able to invade the cells, induced significant modifications of the TJ and adheren proteins. These results indicated that these strains invaded the cell by the crossing of the intestinal barrier after interacting with E-cadherin and with TJ proteins.

A previous work also noted the interaction of the cited proteins with bacteria proteins such as InlA [24]. However, the effect on the TJ and adheren proteins was variable depending on the cell line and the strain. The most notable difference was that the claudin expression level remained invariable with respect to the control in the case of Caco-2 cell line in all the strains (Figure 2A), while it showed significant a decrease in the case of SW480 cell line (ANOVA, *p* < 0.05) (Figure 3A). Besides, ILSI17 was the strain that induced the highest protein expression decrease, followed by ILSI18 (Figure 2 and Figure 3). This fact can explain the high invasion capacity of ILSI17 on SW480 (compared with the rest of cases) (Table 2 and Table 3). The most altered surface protein profile of the SW480 cells probably facilitated the interactions of the *L. monocytogenes* and made the SW480 cell line more susceptible to *Listeria* invasion than the Caco-2 cell. Other authors also observed changes in the gene expression of adhesion proteins and other cell–cell junction proteins in an invasion study of the SW480 cell line [48]. The obtained results seemed to be associated with each cell line structure. Caco-2 forms a cell monolayer that reaches total confluency, differentiated enterocyte, and no accessible basolateral surfaces. According to previous works [24,48], the cited conditions reduce dramatically the invasion capacity of bacteria compared with nonconfluent cell monolayers. On the contrary, the lower differentiated endothelial phenotype and the acquisition of mesenchymal and migratory phenotypes on SW480 result in an exposition of the basolateral surfaces, making the bacteria invasion easier.

### 3.2. Antivirulence Effect of L. monocytogenes Treated with WPP on Caco-2 Cells and SW480 Cell Lines

Phenolic compounds are efficient bacteriostatic agents, and different studies have considered their uses as anti-invasion agents of bacteria [41]. Hence, the treatment of bacteria with WPP, which are very rich in phenolic compounds, could reduce their capacity for cell invasion. To check this hypothesis, the cell lines were inoculated with the four *Listeria* invasive strains after their incubation with WPP-N and WPP-C. (*L. innocua* and E10.652 were excluded from the study due to their lack of invasion capacity). The cell invasion capacity of each WPP-treated *Listeria* strain was expressed as the percentage of invasion with respect to the control group (cell lines inoculated with *Listeria* strains grown in the absence of WPP). *L. innocua*, a nonpathogenic *Listeria* spp. found in similar environments to *L. monocytogenes* [49], was included as a noninvasive and nonpathogenic control.

**Figure 4:**
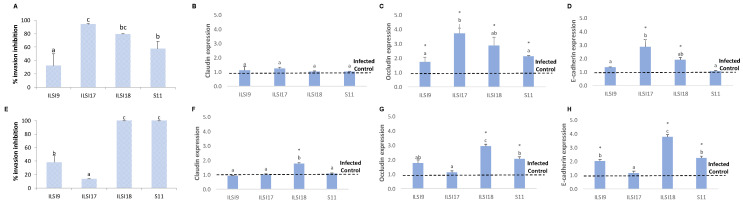
Effect of WPP on the virulence of ILSI9, ILSI17 ILSI18, and S11 *L. monocytogenes* strains on Caco-2 cells. (**A**) In vitro invasion capacity of the *L. monocytogenes* strains treated with WPP-N, expressed as % of inhibition with respect to each control (invasion capacity of each Listeria strain grown in the absence of WPP-N). (**B**–**D**) Relative values of the gene expression of claudin (**B**), occludin (**C**), and E-cadherin (**E**) with respect to the corresponding control (cells infected with each *L. monocytogenes* strain grown in the absence of WPP-N). (**E**) In vitro invasion capacity of the Listeria strains treated with WPP-C, expressed as % of inhibition with respect to each control (invasion capacity of each Listeria strain grown in the absence of WPP-C). (**F**–**H**) Relative values of the gene expression of claudin (**E**), occludin (**F**), and E-cadherin (**G**) with respect to the corresponding control (cells infected with each *L. monocytogenes* strain grown in the absence of WPP-C). Gene expression was assessed by real-time PCR. Results are presented as the means ± SD (*n* = 4). Columns marked with * indicate gene expression values statically different than the respective control (Student’s *t*-test, *p* < 0.05). Letters on each column indicate significant differences among values for each Listeria strain (ANOVA, *p* < 0.05).

**Figure 5:**
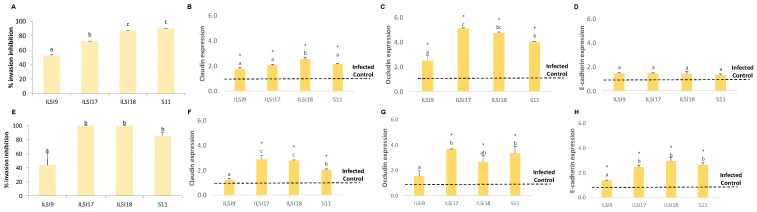
Effect of WPP on the virulence of ILSI9, ILSI17 ILSI18, and S11 *L. monocytogenes* strains on SW480 cells. (**A**) In vitro invasion capacity of *L. monocytogenes* strains treated with WPP-N, expressed as % of inhibition with respect to each control (invasion capacity of each Listeria strain grown in the absence of WPP-N). (**B**–**D**) Relative values of the gene expression of claudin (**B**), occludin (**C**), and E-cadherin (**E**) with respect to the corresponding controls (cells infected with each *L. monocytogenes* strain grown in the absence of WPP-N). (**E**) In vitro invasion capacity of the Listeria strains treated with WPP-C, expressed as % of inhibition with respect to each control (invasion capacity of each Listeria strain grown in the absence of WPP-C). (**F**–**H**) Relative values of the gene expression of claudin (**E**), occludin (**F**), and E-cadherin (**G**) with respect to the corresponding control (cells infected with each *L. monocytogenes* strain grown in the absence of WPP-C). Gene expression was assessed by real-time PCR. The results are presented as the means ± SD (*n* = 4). Columns marked with * indicate gene expression values statically different than the respective control (Student’s *t*-test, *p* < 0.05). Letters on each column indicate significant differences among the values for each Listeria strain (*p* < 0.05).

The results showed a significant effect of WPP on the invasive capacity of the *Listeria* strains (Figure 4 and Figure 5). The incubation with WPP caused an invasiveness inhibition for the four *L. monocytogenes* strains, and in both cases, the Caco-2 and SW480 cell lines, although with quantitative differences between the cell line and WPP.

With respect to the Caco-2 cell line, both WPP, in general, induced a high inhibition of the invasion capacity (Figure 4A,E), although this effect was variable among the strains and WPP. It is remarkable that the most invasive *L. monocytogenes* strain on the Caco-2 cells (ILSI17; Table 2) showed the highest value of inhibition by the treatment with WPP-N (94.4% of inhibition), followed by ILSI18 (79.5% of inhibition), S11 (57.5% of inhibition), and ILSI9 (32.3% of inhibition). These results agree with the observed gene expression levels of occludin and E-cadherin in Caco-2, which were higher when the cells were exposed to *L. monocytogenes* strains incubated with WPP-N with respect to their respective control (cell exposed to the strains not incubated with WPP). Quantitatively, the values were higher, ranging between 3.7 and 1.8 times, in the case of the gene expressions of occludin (Figure 4C). In the case of the gene expression of E-cadherin, only the cells treated with ILSI17 and ILSI18 were significantly different (Student’s *t*-test, *p* < 0.05) and ranged between 2.9 and two times, respectively (Figure 4D). No significative changes were observed in the gene expression of claudin, matching with the respective control (Figure 4B). The last results, together with the invariable levels of claudin expression on the cells invaded with the control *Listeria* strains, suggest that this protein is not involved in the *L. monocytogenes* invasion of Caco-2 cells. Besides, the results noted that WPP incubation reduced the capacity of the bacteria to act on the gene expression of adheren junction and occludin (TJ protein), keeping the cell permeability and reducing the bacteria capacity to infect the cells. It is also remarkable the inhibitory effect of the incubation with WPP-C, raising the 100% of invasion inhibition of the Caco-2 cells for the strains ILSI18 and S11 (Figure 4E). The inhibitory effect agrees, once again, with the gene expression levels of occludin and gene and protein expression of E-cadherin respect to the respective control (invasion with strains incubated in the absence of WPP-C) (Figure 4G,H). Levels of the occludin expression were 2.9 (ILSI18) and 2.1 (S11) times higher, and those of E-cadherin were 3.8 (ILS18) and 2.3 (S11) times higher. Once more, claudin levels were, in general, similar than control cells (Figure 4F). Furthermore, although the inhibition of invasive capacity of ILSI9 was lower than the other strains (ANOVA, *p* < 0.05), the results were also notable since this strain corresponds to the serogroup 1/2b, one of the most related to human cases.

With respect to SW480 cell line, the invasion with *Listeria* strains incubated with both WPP induced high invasiveness inhibition of the four strains (Figure 5A,E), although, once again, this effect was variable among strains and WPP. In general, the inhibition values in this cell line were higher than those obtained in the case of Caco-2 cell line, a fact especially notable comparing with WPP-C effects. Furthermore, the results of the levels of the three protein gene expressions were notably different, especially in the case of claudin levels. A remarkable fact was the total invasive capacity inhibition of ILSI17 (the most invasive *L. monocytogenes* strain, Table 3) incubated with WPP-C on SW480 cell line, while the incubation with WPP-N produced inhibition of 72.6%. The invasion capacity of ILSI18 was also totally inhibited by WPP-C incubation, while the incubation with WPP-N produced inhibition of 87.2%. The incubation of S11 and ILSI9 with WPP-N was some more efficient to reduce invasion capacity (90.4% and 52.3% of inhibition, respectively) than with WPP-C (85.5% and 43.9% of inhibition, respectively). In agreement with these results, in general, incubation with WPP reduced the decrease of gene expression in comparison with the respective control (cell line invaded with strains not incubated with WPP) (Student’s *t*-test, *p* < 0.05); then, some levels of gene expression were higher than those of their control (Figure 5B,C,F–H). The occludin gene expression levels were between 2.6 and 5.1 times higher when *L. monocytogenes* strains were treated with WPP-N respect the corresponding control. These results reflect the involvement of this tight junction protein and the protection of WPP against *L. monocytogenes* invasion of SW480 cells. In this case (SW480 cell line) results showed a significant effect of WPP incubation on the levels of claudin. Gene expression levels of this protein were higher than the control (Student’s *t*-test, *p* < 0.05), noting a higher level of protection, mainly in the case of ILSI17 and ILSI18 incubated with WPP-C (Figure 5B,F). After considering all the commented results together, it is possible to assert that in the case of SW480 cells, the protection of WPP against *L. monocytogenes* invasion involved their interactions on the expressions of both TJ and AJ proteins.

Altogether, the results revealed a significant effect of WPP on the virulence of four *L. monocytogenes* studied strains. This fact could be due to some of the components of WPP, especially some phenolic compounds. Some previous papers reported the anti-listeria activity of various natural products, among them flavonoids [38,50] that can inhibit diverse virulence factors that are not necessary for bacterial viability but are essential for the invasion. One of them is the transpeptidase enzyme sortase A (SrtA) that anchors to the cell wall many of the proteins that are virulence factors required for the *L. monocytogenes* invasion. Some flavonoids as chalcone inhibited SrtA activity [51]. Then, although chalcone levels were not measured in this paper, its presence in WPP is presumable, mainly due to the degradation of anthocyanins during WPP manufacturing. Additionally, different authors pointed out the capacity of monomeric flavan-3-ols, such as catechin, epigallocatechin and their gallate derivatives, to inhibit cyclic dinucleotide metabolism enzymes [48], and others pointed out the inhibitor effect of resveratrol and its derived product on the invasive capacity of *L. monocytogenes* [41]. Both flavan-3-ols and stilbenes, are phenolic compounds present in the two WPP used in this study, and then, it is presumable that these compounds are some of those responsible of the WPP effect on the invasion capacity of the studied *Listeria* strains.

The data suggest that there are different mechanisms involved in the invasion of Caco-2 and SW480 cells by *L. monocytogenes*, probably responding to the degree of differentiation of each cell line and the level of expression and maturation of their cell-cell junctions. In this aspect, the TJ protein occludin is involved in the *L. monocytogenes* invasion of both Caco-2 and SW480 cell lines while claudin is only involved in the invasion of SW480. By the other hand, E-cadherin is also implicated in this invasion, mainly in the cases of the most invasive *Listeria* strains and more clearly in the case of Caco-2 cell line.

## 4. Conclusions

Wine pomace products, with rich contents of the bioactive compounds’ polyphenols, are effective natural products against the invasion of the four invasive *L. monocytogenes* strains studied. The capacity of WPP to reduce or inhibit the invasion of the Listeria strains is associated with their ability to modify the gene expression of adherens and tight junction proteins, which play a notable role in maintaining the integrity of the intestinal cell membrane. Therefore, the results of this study suggest an inhibition of intestinal cell invasion by *L. monocytogenes* treated with a wine pomace product.

## Figures and Tables

**Table 1:** Content of the phenolic compounds (µg/g) found in the wine pomace products (WPPs).

Phenolic Compounds	WPP-N	WPP-C
Gallic acid	266 ± 4	343 ± 1 *
Protocatechuic acid	12.1 ± 0.3	78.2 ± 0.7 *
Gentisic acid	13.4 ± 0.2	41.3 ± 0.4 *
Vanillic acid	8.58 ± 0.20	12.6 ± 0.5 *
Syringic acid	13.4 ± 0.2	1.18 ± 0.06 *
Ethyl gallate	42.3 ± 0.1	23.6 ± 0.3 *
*Total Hydroxybenzoic acids*	*357* *± 3*	500 ± 1 *
p-Coumaric acid	9.93 ± 0.02	10.7 ± 0.1 *
Caffeic acid	32.2 ± 0.5	65.1 ± 0.7 *
*Total Hydroxycinnamic acids*	*42.2 ± 0.4*	*75.9 ± 0.4* *
Catechin	118 ± 2	71.3 ± 0.4 *
Epicatechin	249 ± 1	153 ± 1 *
Epicatechingallate	543 ± 3	672 ± 8 *
Procyanidin B1	186 ± 1	380 ± 5 *
Procyanidin B2	42.7 ± 2.2	33.1 ± 0.2 *
*Total Flavan-3-ols*	*1139 ± 2*	*1311 ± 9* *
t-resveratrol	3.75 ± 0.03	1.81 ± 0.08 *
t-piceid	1.10 ± 0.03	0.68 ± 0.05 *
*Total Stilbenes*	*4.83 ± 0.01*	*2.49 ± 0.03* *
Delphinidin derivatives	221 ± 13	41.2 ± 4.7 *
Cyanidin derivatives	9.37 ± 0.03	3.74 ± 0.16 *
Petunidin derivatives	169 ± 19	11.1 ± 0.1 *
Peonidin derivatives	24.0 ± 1	4.38 ± 0.02 *
Malvidin derivatives	789 ± 65	140 ± 8 *
*Total Anthocyanins*	*1225 ± 98*	201 ± 13 *
TOTAL PHENOLIC COMPOUNDS	2768 ± 93	2090 ± 4 *

Results are expressed as the mean ± standard deviation (*n* = 3). WPP-N: Wine pomace product from the north winery; WPP-C: Wine pomace product of the center winery. * Indicates significative differences (*p* < 0.05) between values.

**Table 2:** Invasion in Caco-2 after *Listeria* growth for 24 and 48 h.

		(cfu/mL)	
	Serogroup	24-h Growth	48-h Growth
*L. innocua*	-	NI	NI
ILSI9	1/2b, 3b, 7	1.1 · 10^3^ ± 7.7 · 10^2^ a	8.3 · 10^2^ ± 3.0 · 10^2^ * α
ILSI17	1/2c, 3c	8.8 · 10^4^ ± 3.6 · 10^3^ c	5.9 · 10^2^ ± 1.0 · 10^1^ * α
ILSI18	1/2a, 3a	6.7 · 10^3^ ± 1.4 · 10^3^ b	1.5 · 10^2^ ± 5.0 · 10^1^ * α
S11	1/2a, 3a	9.4 · 10^2^ ± 4.5 · 10^2^ a	9.6 · 10^2^ ± 3.3 · 10^2^ β
E10.652	1/2a, 3a	NI	NI

NI = not invasion. Significant differences (Student’s *t*-test, *p* < 0.05) between 24 h and 48 h of growth for each strain were indicated with an asterisk (*).Significant differences (ANOVA, *p* < 0.05) between strains and the same hours of growth were indicated with Latin letters (24 h) or Greek letters (48 h).

**Table 3:** Invasion in SW480 after *Listeria* growth for 24 and 48 h.

		(cfu/mL)	
	Serogroup	24-h Growth	48-h Growth
*L. innocua*	-	NI	NI
ILSI9	1/2b, 3b, 7	2.7 · 10^3^ ± 1.7 · 10^3^ a	9.8 · 10^2^ ± 2.6 · 10^2^ * α
ILSI17	1/2c, 3c	3.9 · 10^7^ ± 4.8 · 10^6^ b	5.2 · 10^7^ ± 4.1 · 10^6^ * β
ILSI18	1/2a, 3a	1.3 · 10^3^ ± 2.5 · 10^2^ a	4.3 · 10^1^ ± 0.5 · 10^1^ * α
S11	1/2a, 3a	5.1 · 10^2^ ± 8.2 · 10^1^ a	9.3 · 10^2^ ± 1.3 · 10^2^ * α
E10.652	1/2a, 3a	NI	NI

NI = not invasion. Significant differences (Student’s *t*-test, *p* < 0.05) between 24 h and 48 h of growth for each strain were indicated with an asterisk (*).Significant differences (ANOVA, *p* < 0.05) between strains and the same hours of growth were indicated with Latin letters (24 h) or Greek letters (48 h).
